# *In vitro* co-metabolism of epigallocatechin-3-gallate (EGCG) by the mucin-degrading bacterium *Akkermansia muciniphila*

**DOI:** 10.1371/journal.pone.0260757

**Published:** 2021-12-02

**Authors:** Yun Xia, Xuxiang Zhang, Mingxin Jiang, Hongbo Zhang, Yinfeng Wang, Yuyu Zhang, Robert Seviour, Yunhong Kong

**Affiliations:** 1 School of Agriculture and Life Science, Kunming University, Kunming, China; 2 First Affiliated Hospital of Kunming Medical University, Kunming, China; 3 Microbiology Department, La Trobe University, Bundoora, Victoria, Australia; 4 Dianchi Lake Environmental Protection Collaborative Research Center, Kunming University, Kunming, China; Bangabandhu Sheikh Mujibur Rahman Agricultural University, BANGLADESH

## Abstract

*Akkermansia muciniphila* is a Gram-negative bacterium that resides within the gut mucus layer, and plays an important role in promoting gut barrier integrity, modulating the immune response and inhibiting gut inflammation. Growth stimulation of *A*. *muciniphila* by polyphenols including epigallocatechin-3-gallate (EGCG) from difference sources is well-documented. However, no published *in vitro* culture data on utilization of polyphenols by *A*. *muciniphila* are available, and the mechanism of growth-stimulating prebiotic effect of polyphenols on it remains unclear. Here *in vitro* culture studies have been carried out on the metabolism of EGCG by *A*. *muciniphila* in the presence of either mucin or glucose. We found that *A*. *muciniphila* did not metabolize EGCG alone but could co-metabolize it together with both these substrates in the presence of mineral salts and amino acids for mucin and protein sources for glucose. Our metabolomic data show that *A*. *muciniphila* converts EGCG to gallic acid, epigallocatechin, and (-)-epicatechin through ester hydrolysis. The (-)-epicatechin formed is then further converted to hydroxyhydroquinone. Co-metabolism of *A*. *muciniphila* of EGCG together with either mucin or glucose promoted substantially its growth, which serves as a further demonstration of the growth-promoting effect of polyphenols on *A*. *muciniphila* and provides an important addition to the currently available proposed mechanisms of polyphenolic prebiotic effects on *A*. *muciniphila*.

## Introduction

*Akkermansia muciniphila* is a Gram-negative bacterium that resides within the mucus layer covering the gut, and represents 3–5% of the gut microbiota in healthy adults [1,2]. Its genome encodes a cluster of mucin-degrading enzymes, thus enabling it to use mucin as its sole source of carbon and nitrogen [3,4]. *A*. *muciniphila* plays an important role in promoting gut barrier integrity, modulating the host immune response, inhibiting inflammation and enriching butyrate-producing bacteria [5]. Its abundance is correlated inversely with presence of metabolic diseases including diabetes and obesity [6], ulcerative colitis [7], HIV [5] and cancers [8–10]. Therefore, *A*. *muciniphila* has been proposed as an emerging “gatekeeper of the gut” and a “next-generation beneficial microbe” [11].

Enhancing the abundance of *A*. *muciniphila* in the intestinal microbiota has been shown to contribute towards the prevention and treatment of metabolic diseases [5,12,13], and dietary intervention has been effective in maintaining an intestine homeostasis with a high ratio of *A*. *muciniphila* [14]. Epidemiological and animal feed intervention studies have established a solid association between the consumption of polyphenol-rich foods or beverages and subsequent enrichment of *A*. *muciniphila*, together with associated preventive influences on various metabolic diseases [15–19]. However, most of these studies are based on positive correlations between the consumption of polyphenol-rich foods or beverages and subsequent enrichment of *A*. *muciniphila*. No published *in vitro* culture data on *A*. *muciniphila* utilization of polyphenols are available, and so the promoting mechanism remains unclear.

Polyphenols include the flavonoids, phenolic acids, stilbenes and lignans. More than 9000 different structures have been described for the flavonoid group alone [20]. Of these, the prebiotic effects of epigallocatechin-3-gallate (EGCG), a natural phenolic compound found in many plants, especially in green tea, have been studied. Like the majority of the polyphenols (90–95%), EGCG is poorly bioavailable in the small intestine and is transported to the large intestine where it undergoes biotransformation by the gut microbiota [21]. EGCG has been suggested to improve therapeutic outcomes for diseases including cancers, inflammations, HIV, diabetes, obesity, neurodegenerative diseases such as Alzheimer’s disease and Parkinson’s disease (reviewed by Yang et al. [22]). EGCG is known to modify the gut microbiota and increase the abundance of *A*. *muciniphila*, leading to improved gut dysbiosis and barrier integrity [23–27]. However, as with most other polyphenolic compounds, its stimulatory mechanisms in *A*. *muciniphila* are still largely unknown. In a previous metaproteomic study, we showed that aqueous extracts of Pu-er raw tea stimulated expression of key metabolic genes of *A*. *muciniphila* and increased its abundance in the cecum of rats [19]. In this study, we have investigated further how *A*. *muciniphila* metabolizes EGCG using an *in vitro* culture approach. Understanding the mechanism of action of dietary polyphenols is considered likely to be the key for designing future nutritional compositions of food leading to optimal health benefits [28].

## Materials and methods

### Chemicals

EGCG, (-)-catechin gallate, gallic acid, gallocatechin, (-)-epigallocatechin, Fe_4_(P_2_O_7_)_3_, vitamin K (Yuanyue Biology, Shanghai, China); KH_2_PO_4_, Na_2_HPO_4_, NH_4_Cl, NaCl, MgCl_2_.6H_2_O, CaCl_2_, NaHCO_3_, Na_2_S.7–9H_2_O, FeCl_2_, H_3_BO_4_, ZnCl_2_, CuCl_2_, MnCl_2_, CoCl_2_, NiCl_2_, HCl, Na_2_SeO_3_, Na_2_WO_4_, Na_2_MoO_4_, NaOH and starch (Tianjin Fengchuan Chemical Reagent Technologies Co., Ltd, Tianjin, China); hog gastric mucin (Type III), biotin, niacin, pyridoxine, riboflavin, thiamine, cyanocobalamin, p-aminobenzoic acid, and pantothenic acid, hydroxyhydroquinone, 3,4-dihydroxybenzaldehyde, resazurin (Sigma, Shanghai, China); peptone, yeast extract, glucose, sodium pyruvate, arginine, sodium succinate, L-cysteine HCl, haemin, sodium thioglycolate, Cellobiose (Meilunbio, Dalian, China); paraformaldehyde, SDS, formamide (Biotopped, Bejing, China) were all of analytical grade, with mass fractions ≥ 98%.

### Bacterial strain, culture media and growth conditions

*A*. *muciniphila* MucT (= DSM 22959T) was obtained from the China General Microbiological Collection Center, Beijing, China. The bacterium was kept in agar plates of DSM medium 1203a. For experiments, a single colony of *A*. *muciniphila* was transferred either into the BS broth of [29] for EGCG and mucin metabolic trials or the BS broth supplemented with 2% (w/v) peptone and yeast extract (modified BS broth hereafter) for EGCG and glucose metabolic trials. EGCG was used as a supplement at 0, 150, 350 and 500 mg L^-1^ final concentrations, obtained from a 100× concentrated filter-sterilized EGCG stock solution. Mucin, purified by ethanol precipitation as described by Miller and Hoskins [30], was added at 0.25% (w/v), while glucose was added at 2 g L^-1^ final concentration.

The BS medium contained (g L^-1^): KH_2_PO_4_ 0.4; Na_2_HPO_4_ 0.53; NH_4_Cl 0.3; NaCl 0.3; MgCl_2_.6H_2_O 0.1; CaCl_2_ 0.11; 1 mL alkaline trace element solution, 1 mL acid trace element solution and 1 mL vitamin solution described by Stam et al. [31]; resazurin 0.0005; NaHCO_3_ 4; Na_2_S.7–9H_2_O 0.25. The resazurin was added to the medium as an indicator of medium anaerobiosis. The pH was adjusted to 7.0 before sterilization, and the vitamin solution was filter-sterilized, while all other ingredients were autoclaved (121°C for 30 min). The acid trace element solution contained (millimolar): FeCl_2_ 7.5; H_3_B0_4_ 1; ZnCl_2_ 0.5; CuCl_2_ 0.1; MnCl_2_ 0.5; CoCl_2_ 0.5; NiCl_2_ 0.1; and HCl 50, while the alkaline trace element solution contained (millimolar): Na_2_SeO_3_ 0.1; Na_2_WO_4_ 0.1; Na_2_MoO_4_ 0.1; and NaOH 10 and the vitamin solution contained (g L^-1^): biotin 0.02; niacin 0.2; pyridoxine 0.5; riboflavin 0.1; thiamine 0.2; cyanocobalamin 0.1; p-aminobenzoic acid 0.1; and pantothenic acid 0.1.

*A*. *muciniphila* was grown under anaerobic conditions for 24 h at 37°C in 2.5 L anaerobic jars (Mitsubishi Gas Chemical Company INC, China), each containing one AnaeroPack produced by the same company. The AnaeroPack is a disposable oxygen-absorbing and carbon dioxide-generating, sealed porous sachet for use in anaerobic jars. There is no need to add water or to use a palladium catalyst. The contents of the sachet become activated immediately in contact with oxygen. Once the AnaeroPack is placed in the anaerobic jars, the oxygen level is reduced to < 1% in 30 min. At the same time, carbon dioxide is generated to a concentration of 18% in approximately 12 min [30]. The evaluation study by Delaney and Onderdonk [32] has shown that the AnaeroPack system is an excellent alternative to established methods including conventional anaerobic chambers for generating an environment for anaerobic culture incubation. Cells were then harvested by centrifugation (6,000 × g, 10 min, 5°C), washed twice with 0.9 g/100 mL NaCl solution and suspended in the same solution adjusted to an OD600 0.6 before being used as the experimental inoculum. A 9 g L^-1^ NaCl solution was used for control purpose.

#### Measurement of growth

Growth of *A*. *muciniphila* was determined spectrometrically by measuring the optical density (OD) at 600 nm of samples taken at regular intervals following inoculation. The relative growth yield of *A*. *muciniphila* on either mucin and glucose with EGCG supplementation was calculated based on its OD600 according the method of Crittenden et al. [33], using the following formula:

Relativegrowthyield=[(A–B)/(C–B)]×100%

where A is the net change in OD600 of culture grown on either mucin or glucose plus EGCG, B is the net change in OD600 of culture grown with only EGCG, and C is the net change in OD600 of culture grown on either mucin or glucose. The growth yields of *A*. *muciniphila* from growth on either glucose or mucin plus 500 mg L^-1^ EGCG equaled 100%.

### Experimental conditions for studying co-metabolism of EGCG by *A*. *muciniphila*

To investigate metabolism of EGCG by *A*. *muciniphila* in the presence of mucin, 0.1 mL of harvested cells (incubated in the same medium) adjusted to an OD600 of 0.6 was mixed with 4.9 mL BS broth with or without mucin, and supplemented with EGCG at 0, 150, 350 and 500 mg L^-1^ final concentrations in 10 mL serum bottles (bottom diameter 2 cm) with aluminum caps. These were incubated anaerobically in the anaerobic jars at 37°C for 24–72 h. The resazurin indicator in the BS broth became colorless within 2 h, indicating anaerobiosis was achieved. Six replicates were included for each treatment and OD600 values were determined spectrophotometrically. The cells were collected by centrifugation (6,000 × g, 10 min, 5°C), washed 3 times in ice-cold PBS solution before being frozen immediately in liquid nitrogen for subsequent metabolomic analysis (see below) and finally fixed in 4% paraformaldehyde for fluorescence *in situ* hybridization (FISH) [34]. The supernatants were analyzed immediately using HPLC to determine EGCG concentrations. Experimental protocols for investigating *A*. *muciniphila*’s metabolism of EGCG with glucose were the same as detailed above except that the modified BS broth was used.

### HPLC quantification of EGCG

Supernatants were filtered through 0.45 μm membrane filters and the filtrates analyzed immediately. Analyses were performed with a HPLC (Waters e2659, Beijing, China) with a Thermo Hypersil GOLD C18 column (4.6 mm × 250 mm, 5 μm) (Jingouya Biotech. Co., Beijing, China). A binary gradient elution protocol consisting of solvent A (9% acetonitrile and 2% acetic acid) and solvent B (80% acetonitrile and 2% acetic acid) was applied as follows: 100% A (0–10 min); 100% A to 68% A and 32% B (10–25 min); 68% A and 32% B (26–35 min); 68% A and 32% B to 100% A (36–35 min); 100% A (36–45 min). The column temperature was maintained at 35°C and the injection volume was 10 μL. The UV detection wavelength was set at 278 nm, and EGCG identified by its retention time against EGCG standards.

### Metabolomic analysis

Aliquots of 50 mg pelleted cells were suspended in 400 μL methanol:water (4:1, v/v) solution and then exposed to a high throughput tissue crusher (Wonbio-96c, Shanghai Wanbo Biotechnology Co., LTD, Shanghai, China) at 40 KHz and 5°C for 30 min, and allowed to settle at –20°C for 30 min. Cell debris was collected by centrifugation (13,000 × g, 4°C for 15 min) and dried in a N_2_ stream after the supernatants were decanted. It was then resuspended in 120 μL acetonitrile:water (1:1, v/v), and again extracted with the tissue crusher under the same conditions as above for 5 min followed by centrifugation (13,000 × g, 5 min, 4°C) and the supernatants were again collected. These were transferred carefully to sample vials for subsequent LC-MS/MS analysis and later HPLC analysis using standard polyphenolic compounds.

Chromatographic separation of cell free metabolites was performed on a ExionLC^TM^AD system (AB Sciex, USA) equipped with an ACQUITY UPLC BEH C18 column (2.1 × 100 mm, 1.7 μm, Waters, Milford, USA) at a flow rate of 0.4 mL min^-1^. The injection volume was 5 μL. A gradient program using 0.1% formic acid in water (Solvent A) and 0.1% formic acid in acetonitrile:isopropanol (1:1, v/v) (Solvent B) was applied as follows: 95% A and 5% B to 80% A and 20% B (0–3 min); 80% A and 20% B to 5% A and 95% B (3–9 min); 5% A and 95% B to 5% A and 95% B (9–13 min); 5% A and 95% B to 95% A and 5% B (13–13.1 min), 95% A and 5% B (13.1–16 min). Column temperature was maintained at 40°C.

The UPLC system was coupled to a quadrupole-time-of-flight mass spectrometer (Triple TOFTM5600+, AB Sciex, USA) equipped with an electrospray ionization source operating in positive and negative mode. Optimal conditions were set as follows: source temperature, 500°C; curtain gas, 30 psi; both Ion Source GS1 and GS2, 50 psi; ion-spray voltage floating, −4000 V in negative mode and 5000 V in positive mode, respectively; de-clustering potential, 80 V; a collision energy, 20–60 V rolling for MS/MS. Data acquisition was performed with the Data Dependent Acquisition mode. Detection was carried out over a mass range of 50–1000 m/z.

Raw data from UPLC-TOF/MS analyses were imported into the Progenesis QI 2.3 (Nonlinear Dynamics, Waters, USA) software for peak detection and alignment. Metabolic features that were detected in at least 80% of the samples were recorded. To reduce possible biases arising from sample preparation and equipment instability, key metabolic features were normalized as follows. Those with relative standard deviations from their quality control of > 30% were discarded and the resulting data were then log transformed before matching with the Human metabolome database (HMDB) (http://www.hmdb.ca/) and Metlin database (https://metlin.scripps.edu/) for metabolite identification. The mass tolerance between the measured m/z values and the exact mass of the components of interest was ± 10 ppm. For metabolites having MS/MS confirmation, only those with MS/MS fragment scores of > 30 were considered as being identified with confidence. Differentiating metabolites between different treatment groups used a VIP (variable importance in the projection) value > 1 and a P value < 0.05. Metabolomics data have been deposited in the EMBL-EBI MetaboLights database [35] with the identifier MTBLS2798. The complete dataset can be accessed at https://www.ebi.ac.uk/metabolights/MTBLS2798.

HPLC analyses of EGCG metabolites of *A*. *muciniphila*’s cell extracts using standard polyphenolic compounds were performed with the HPLC setups used to measure EGCG concentrations in the BS broth. A binary gradient elution protocol consisting of solvent A (2% acetic acid containing 0.2% EDTA) and solvent B (80% acetonitrile) was applied as follows: 95% A and 5% B (0–10 min); 95% A to 60% A and 5% B to 40% B (11–15 min); 60% A and 40% B (16–20 min); 95% A and 5% B (21 min); 95% A and 5% B (22–30 min). The column temperature was maintained at 35°C and the injection volume was 10 μL. The UV detection wavelength was set at 278 nm, and EGCG metabolites identified by their retention times against the standards.

### FISH analyses

Purity of *A*. *muciniphila* cultures was examined by FISH using the Cy3-labeled probe MUC-1437 (5’-CCT TGC GGT TGG CTT CAG AT-3’) designed by Derrien et al. [36] to target specifically the 16S rRNA sequence of *A*. *muciniphila* against all the bacterial cells staining positively with DAPI (4’,6-diamidino-2-phenylindole) [100 μL (0.003 mg mL^-1^) for 10 min]. All probes were purchased from Sangon Biotech (Shanghai, China). FISH was carried out according to the procedure described by Kong et al. [34], with a formamide concentration of 30%. At least 10 sets of the same view of Cy3 and DAPI images for each individual culture taken using an epifluorescence microscope (Olympus BX53, Nanjing, China) were examined. *A*. *muciniphila* cultures containing any cells staining with DAPI but not hybridizing with the FISH probe MUC-1437 on any of the images examined were discarded.

### Statistical analyses

Significances between relative growth yield based on OD600 values of the different treatment groups were calculated using the Kruskal-Wallis test with SPSS (version 16.0). Significant differences were established at P < 0.05. A multivariate statistical analysis of the metabolomic data was performed using ropls (Version1.6.2, http://bioconductor.org/packages/release/bioc/html/ropls.html) from the R package from Bioconductor on a Majorbio Cloud Platform (https://cloud.majorbio.com). Principle component analysis (PCA) using an unsupervised method was applied to obtain an overview of the metabolic data and general clustering trends, and any outliers were visualized. All metabolite variables were scaled to unit-variances prior to performing the PCA. Orthogonal partial least squares discriminant analysis (OPLS-DA) was used for statistical analysis to determine global metabolic changes between comparable groups. All metabolite variables were scaled to Pareto Scaling prior to conducting the OPLS-DA. The model validity was evaluated from model parameters R2 and Q2, which provide information for the interpretability and predictability respectively of the model, and avoid the risk of over-fitting. VIP values were calculated with the OPLS-DA model. P values were estimated with paired Student’s t-test on single dimensional statistical analysis. Differential metabolites between different treatment groups were determined with a VIP value > 1 and a P value < 0.05.

## Results

### Co-metabolism of EGCG and mucin by *A*. *muciniphila*

**Fig 1:**
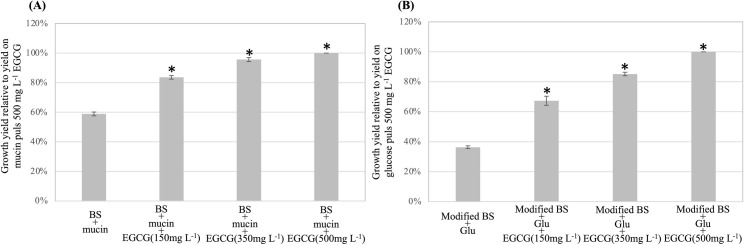
Effects of EGCG supplementation on the growth of *Akkermansia muciniphila*. Relative growth yields of *A*. *muciniphila* cultivated in the mucin BS broth **(A)** and the glucose supplemented modified BS broth **(B)** both supplemented with or without EGCG at 150, 350 and 500 mg L^-1^ final concentrations. The growth yield of *A*. *muciniphila* cultivated with mucin or glucose supplemented with 500 mg L^-1^ EGCG was calculated at 100%. * indicates significant difference (P < 0.05) to the control.

The BS broth of Derrien et al [26] which contains only mineral solutions and amino acids was used to investigate metabolism of EGCG in the presence of mucin or glucose by *A*. *muciniphila*. EGCG was co-metabolized with mucin by *A*. *muciniphila*, which grew better when both EGCG and mucin were present in the BS broth than with only mucin. After 24 h, the relative growth yields of *A*. *muciniphila* in the BS broth with mucin and EGCG supplemented at final concentrations of 150, 350 and 500 mg L^-1^ were all dose-dependently significantly (P < 0.05) higher (1.42, 1.62 and 1.70 times respectively) than the corresponding controls with no EGCG supplementation (Fig 1A). *A*. *muciniphila* could not metabolize EGCG in the BS broth in the absence of mucin. No growth of *A*. *muciniphila* was observed after 24, 48 and 72 h incubation in the BS broth in the presence of only EGCG supplemented at the three different levels used. Microscopic observation showed that the cell morphology of *A*. *muciniphila* in the incubations with EGCG did not differ from that with no EGCG (control), with cells in both being oval-shaped.

### Co-metabolism of EGCG and glucose by *A*. *muciniphila*

*A*. *muciniphila* could also metabolize EGCG in the presence of glucose (Fig 1B) in the BS broth supplemented with peptone and yeast extract (modified BS broth). Moreover, supplementation with EGCG further promoted its growth. Thus, after 24 h the OD600 values of the modified BS broth supplemented with glucose at 2 g L^-1^ final concentration and EGCG at 150, 350 or 500 mg L^-1^ final concentration were dose-dependently significantly (P < 0.05) higher (1.87, 2.34 and 2.77 times respectively) than those of the corresponding controls with no EGCG supplementation (Fig 1B).

### Extent of EGCG utilization by *A*. *muciniphila* in the presence of either mucin or glucose

The EGCG concentrations in the BS broth with and without mucin, and the modified BS broth with and without glucose were examined by HPLC, and the results obtained are summarized in S1 Table. After 24 h, 91.2%, 83.9% and 72.2% of EGCG supplemented in the mucin BS broth at a final concentration of 150, 350 and 500 mg L^-1^ respectively had been utilized. Similarly, 88.5%, 78.3% and 68.5% of EGCG supplemented in the modified BS glucose broth were utilized by *A*. *muciniphila*. In contrast, initial concentrations of EGCG in the BS broth containing no mucin or glucose did not change markedly after 24, 48 and 72 h incubation (S1 Table).

### Products of EGCG metabolism by *A*. *muciniphila*

**Fig 2:**
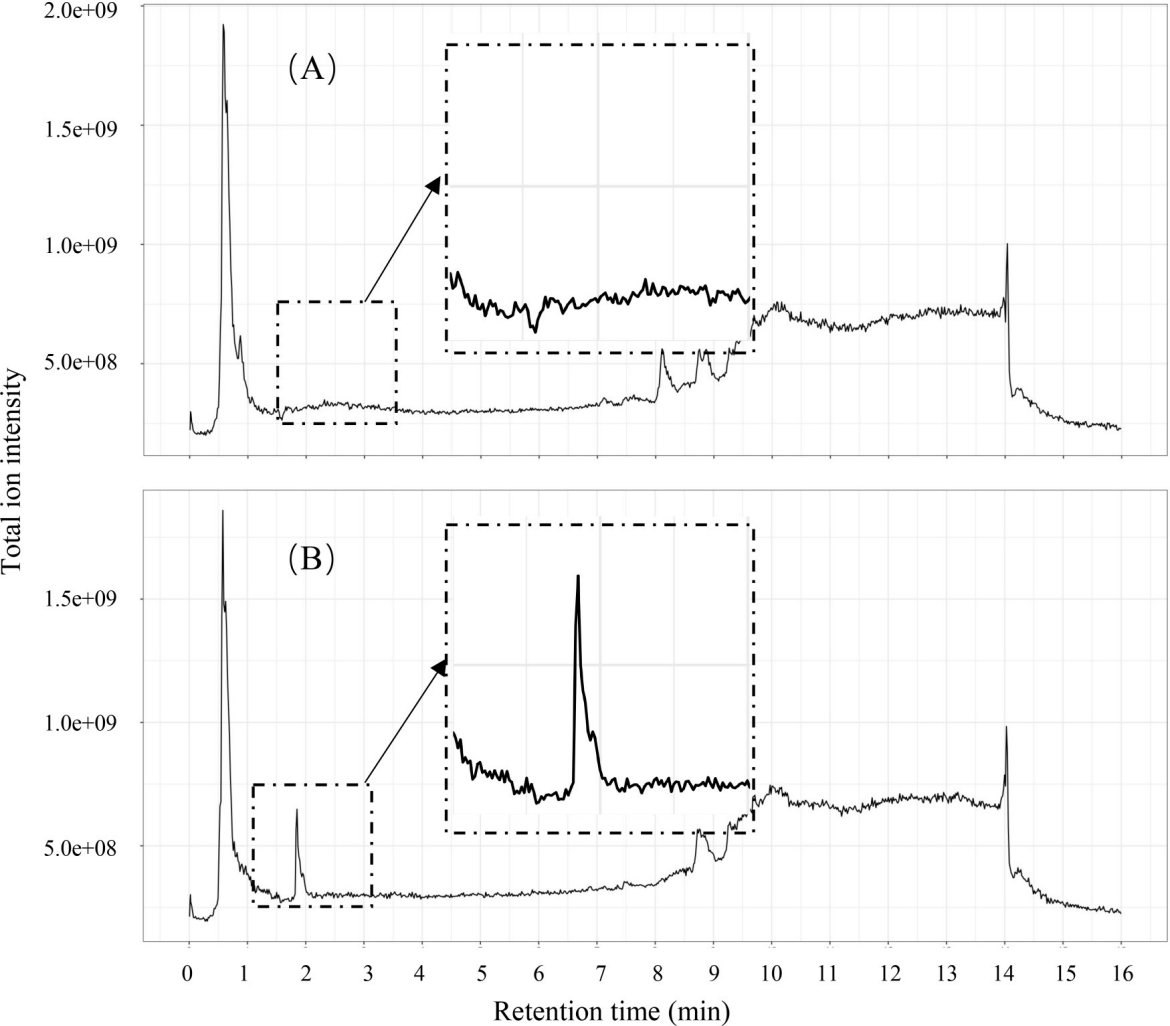
Total ion chromatograms (Ionization–mode) of *Akkermansia muciniphila* cultivated in the mucin BS broth supplemented with (B) or without (A) EGCG at a final concentration of 350 mg L^-1^.

To investigate the composition of metabolites and the metabolic pathways involved in EGCG degradation by *A*. *muciniphila*, metabolomic analyses were carried out to identify EGCG degradation products in both the mucin supplemented BS broth and glucose supplemented modified BS broth with or without EGCG (350 mg L^-1^). Representative total ion chromatograms of *A*. *muciniphila* are shown in Fig 2. We found that EGCG supplementation changed considerably the composition of the *A*. *muciniphila* metabolic biproducts. In total, 3550 (1625 with ionization + mode and 1925 with ionization–mode) and 1596 (808 with ionization + mode and 787 with ionization–mode) spectrometric features were generated from *A*. *muciniphila* cultivated in the mucin BS broth and the glucose supplemented modified BS broth, respectively.

**Fig 3:**
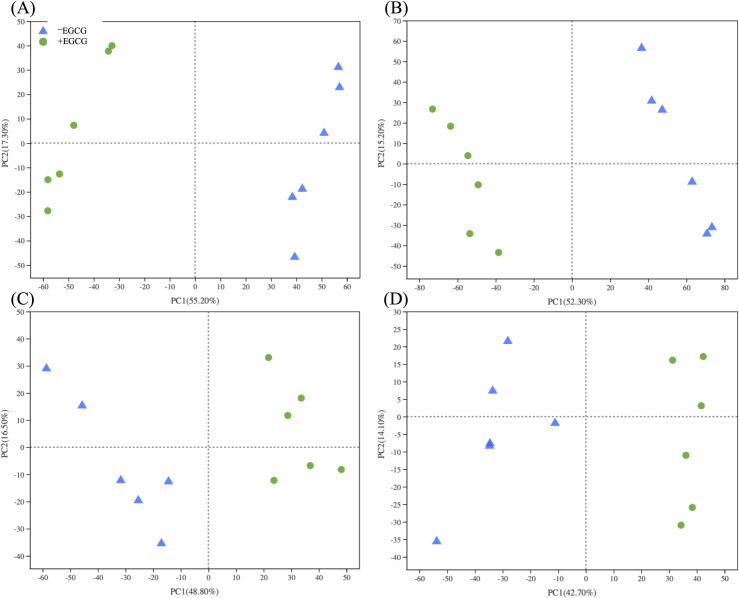
Grouping of the total metabolites of *Akkermansia muciniphila* cultivated in the mucin BS broth (6 replicates) supplemented with or without EGCG at a final concentration of 350 mg L^-1^ based on principle component analysis.

Metabolites identified from *A*. *muciniphila* cultures all showed a clear segregation between those grown with or without EGCG (Fig 3), with 152 and 107 different catabolic metabolites identified respectively in these two experiments. Of the metabolites identified, 25 and 28 respectively were produced by *A*. *muciniphila* grown with mucin (Table 1) and with glucose (Table 2). These included a range of polyphenolic products [37] including catechin gallate, gallocatechin, epigallocatechin, gallic acid, esculetin, hydroxyhydroquinone, 3,4-dihydroxybenzaldehyde, and umbelliferone (Tables 1 and 2).

**Table 1:** Differential metabolites produced by *Akkermansia muciniphila* cultivated in the mucin BS broth supplemented with or without EGCG at a final concentration of 350 mg L^-1^.

No.	Differential metabolite	Ionization mode	Retention time (min)	Mean peak intensity^a^ (Standard deviation)		P value
				With EGCG	Without EGCG	
**1**	Glycerol 1-(5-hydroxydodecanoate)	+	0.9023	–	–	9.73E-07
**2**	9(S)-HODE	+	8.0109	–	–	5.18E-10
**3**	13’-Carboxy-gamma-tocotrienol	+	1.3142	–	–	1.34E-10
**4**	Squamotacin	+	10.1060	–	–	1.65E-04
**5**	Austalide J	+	10.6111	–	–	1.90E-04
**6**	Sagittariol	+	8.0109	–	–	3.80E-09
**7**	N-Hexadecanoylpyrrolidine	+	7.1424	–	–	1.81E-05
**8**	N-(14-Methylhexadecanoyl)pyrrolidine	+	7.1226	–	–	3.66E-04
**9**	16-Hydroxy hexadecanoic acid	+	4.6790	–	–	4.29E-07
**10**	2-(1-Propenyl)-delta1-piperideine	+	4.2273	–	–	3.19E-08
**11**	Ethyl 3-hydroxydodecanoate	+	3.5493	–	–	6.90E-08
**12**	9-Hydroxy-4-methoxypsoralen 9-glucoside	+	1.5778	–	–	3.66E-18
**13**	Cysteinyl-Hydroxyproline	+	0.9023	–	–	5.07E-11
**14**	Epigallocatechin gallate^b^	−	2.2993	516885.5 (± 228192.5)	5.7 (± 2.8)	5.42E-13
**15**	(-)-Catechin gallate^b^	−	2.6610	830320.7 (± 86598.3)	13.4 (± 31.3)	1.11E-18
**16**	Gallic acid^b^	−	1.3470	257686.6 (± 35558.7)	340.3 (± 70.9)	1.35E-13
**17**	Esculetin^b^	−	2.7059	14141.2 (± 1261.5)	205.7 (± 48.9)	1.16E-11
**18**	6-Methylthiopurine 5’-monophosphate ribonucleotide	−	3.0225	–	–	1.86E-08
**19**	1-Heptadecanoylglycerophosphoethanolamine	−	10.3677	–	–	1.25E-04
**20**	2-Dodecylbenzenesulfonic acid	−	9.2458	–	–	3.17E-05
**21**	Isoeugenitol	−	2.9769	–	–	1.19E-12
**22**	Epitheaflavic acid 3’-gallate	−	2.7059	–	–	2.97E-09
**23**	7-deshydroxypyrogallin-4-carboxylic acid	−	2.5026	–	–	1.04E-08
**24**	Ethyl aconitate	−	2.0962	–	–	1.26E-12
**25**	Hydroxyhydroquinone^b^	−	1.3470	7296.3 (± 980.8)	492.1 (± 22.8)	1.86E-12

^a^ only relative peak intensities of polyphenolic compounds are listed.

^b^ indicates polyphenolic compounds.

**Table 2:** Differential metabolites produced by *Akkermansia muciniphila* cultivated in the modified BS broth containing glucose (2 g L^-1^) and supplemented with or without EGCG at a final concentration of 350 mg L^-1^.

No.	Differential metabolite	Ionization mode	Retention time (min)	Mean peak intensity^a^ (Standard deviation)		P value
				With EGCG	Without EGCG	
**1**	6-Oxopiperidine-2-carboxylic acid	+	0.7694	–	–	3.357E-06
**2**	3,4-Dihydroxybenzaldehyde^b^	+	1.8554	3095164.4 (± 1037699.6)	789.2 (± 1031.9)	2.128E-06
**3**	(+)-Gallocatechin^b^	+	1.8554	3823219.3 (± 1268477.9)	211.5 (± 494)	2.504E-08
**4**	2-(1,2-Diamino-1-propenyl)phenol	+	1.3211	–	–	1.374E-10
**5**	6,8-dihydroxy-7-methoxy-2H-chromen-2-one	+	1.4183	–	–	1.56E-05
**6**	Diphenylurea	+	2.1467	–	–	1.60E+05
**7**	Isorhamnetin 4’-O-glucuronide	+	2.2925	–	–	3.925E-06
**8**	Cyclo(L-Phe-L-Pro)	+	2.6000	–	–	0.01195
**9**	Flazine	+	3.4900	–	–	0.003548
**10**	Dehydroxymethylflazine	+	3.9754	–	–	0.004441
**11**	Dihydrodaidzein	+	2.4543	–	–	0.001895
**12**	Queuosine	+	2.3572	–	–	6.24E-05
**13**	Pilosine	+	1.8878	–	–	2.21E-05
**14**	Nicotine glucuronide	+	1.7745	–	–	1.79E-05
**15**	Feruloylagmatine	+	1.7583	–	–	1.352E-06
**16**	P-Coumaroylagmatine	+	1.6611	–	–	3.353E-09
**17**	Butyl (S)-3-hydroxybutyrate glucoside	+	1.5641	–	–	0.002548
**18**	Tuberoside J	+	1.2238	–	–	0.004006
**19**	Hordenine	+	0.9966	–	–	0.001913
**20**	Arginyl-Proline	+	0.9641	–	–	0.003312
**21**	Thiodiacetic acid	+	0.6073	–	–	0.004467
**22**	4-Guanidinobutanal	+	0.6073	–	–	0.001119
**23**	(-)-Catechin gallate^b^	⎻	2.3330	94124.2 (± 35598.9)	0	0.001347
**24**	Epigallocatechin^b^	⎻	1.8509	103256.6 (± 42760.8)	0	0.00248
**25**	Gallic acid^b^	⎻	0.9866	91155.4 (± 21114.4)	1.5 (± 3.4)	5.171E-10
**26**	Umbelliferone^b^	⎻	1.8509	14838.6 (± 5854.6)	0	0.002169
**27**	Avicularin	⎻	1.8509	–	–	0.0008627
**28**	Hydroxyhydroquinone^b^	⎻	1.8509	251637.8 (± 93015.2)	857.2 (± 402.8)	5.361E-10

^a^ only relative peak intensities of polyphenolic compounds are listed.

^b^ indicates polyphenolic compounds.

**Fig 4:**
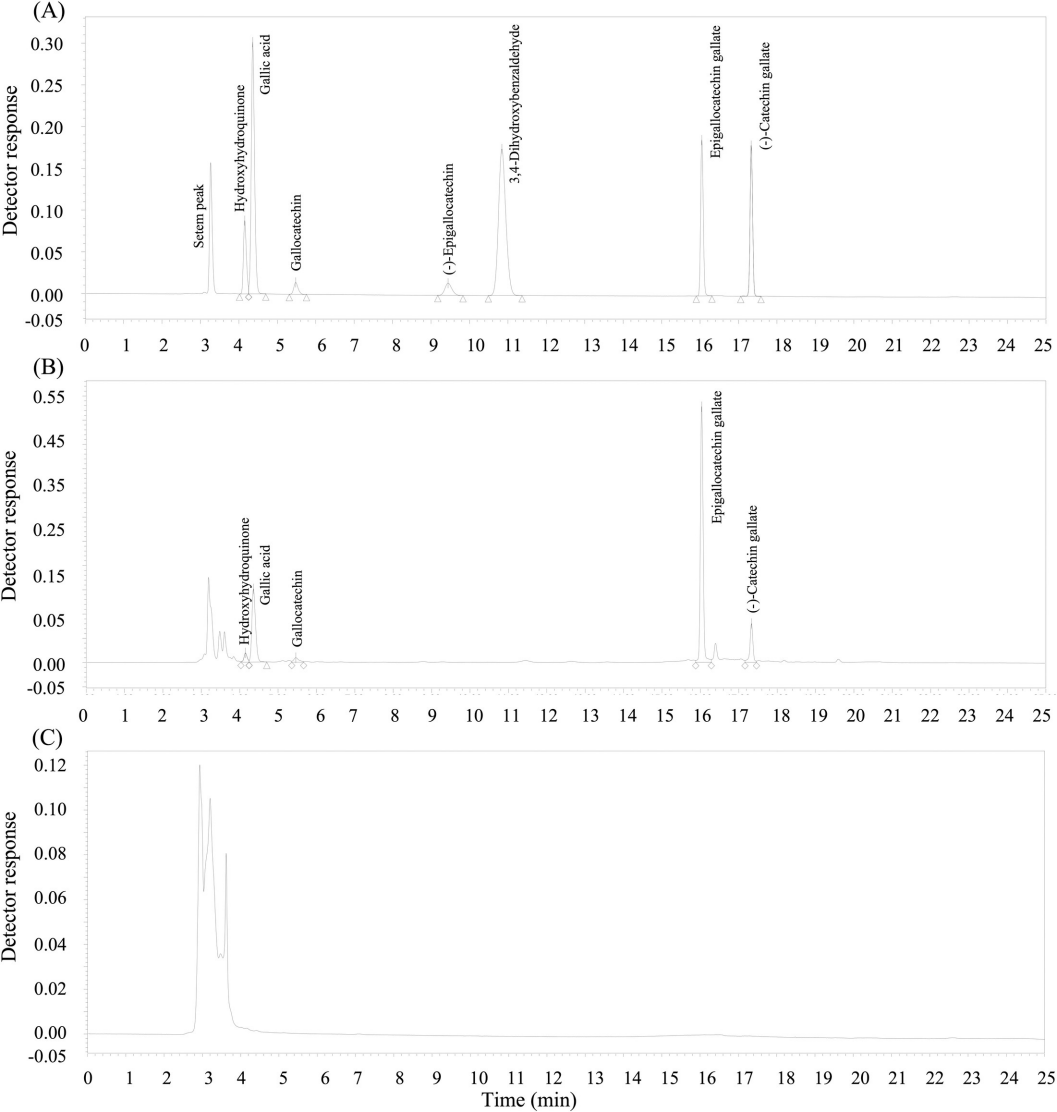
HPLC spectra of polyphenolic compounds produced by *Akkermansia muciniphila* cultivated with the mucin BS broth. (A) HPLC spectrum of standard polyphenolic chemicals; (B) HPLC spectrum of cell extracts of *A*. *muciniphila* cultivated with mucin BS broth with EGCG (350 mg L^-1^); (C) HPLC spectrum of cell extracts of *A*. *muciniphila* cultivated with mucin BS broth without EGCG (350 mg L^-1^).

**Fig 5:**
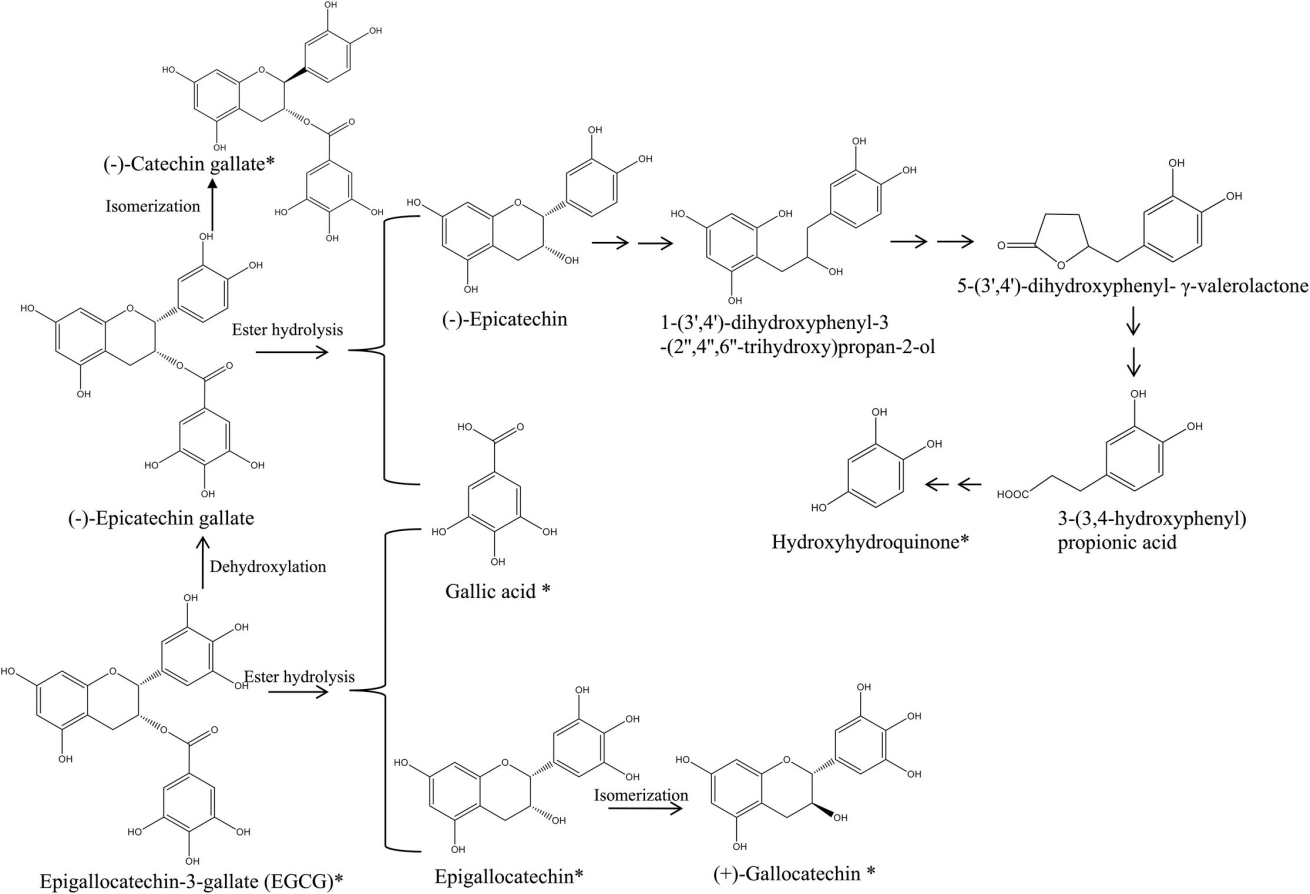
Proposed EGCG metabolic pathways of *Akkermansia muciniphila* based on the polyphenolic compounds produced by *A*. *muciniphila* cultivated in the mucin BS broth and glucose supplemented modified BS broth both supplemented with EGCG at a final concentration of 350 mg L^-1^.

The identification of these polyphenolic metabolites of EGCG identified with LC-MS/MS was confirmed using HPLC with appropriate standards of (-)-catechin gallate, gallocatechin, (-)-epigallocatechin, gallic acid, hydroxyhydroquinone, and 3,4-dihydroxybenzaldehyde which had earlier been either identified by LC-MS/MS at relatively high abundances in *A*. *muciniphila* cells, or were present in *A*. *muciniphila* cells incubated in the BS medium plus EGCG and mucin and the modified BS medium plus EGCG and glucose (Fig 4, Tables 1 and 2). As shown in a representative HPLC spectrum (Fig 4), hydroxyhydroquinone, gallic acid, gallocatechin, (-)-catechin gallate were identified (S2 Table) in *A*. *muciniphila* cells incubated with EGCG (Fig 4B) but not in those incubated without it (Fig 4C, S2 Table). Proposed degradation pathways in *A*. *muciniphila* for EGCG based on these data are shown in Fig 5.

## Discussion

Growth stimulation of *A*. *muciniphila* by polyphenols from a range of sources has been well-documented in animal models and epidemiological studies [24–26]. Thus, Isnard et al. [38] investigated the effects of metformin on the weight and gut microbiota of 23 nondiabetic people with HIV in a Lilac pilot trial. They found that metformin changed the gut microbiota composition by increasing the abundance of anti-inflammatory bacteria including butyrate-producing species and the protective *A*. *muciniphila*. Grajeda-Iglesias et al. [39] reported that oral administration of *A*. *muciniphila* impacted the metabolism of mice, causing an increase in levels of short-chain fatty acid, 2-hydroxybutyrate, bile acids, spermidine and other polyamines in the gut and in the liver. All these metabolites have been associated with affecting human health. In an ongoing project (https://clinicaltrials.gov/ct2/show/NCT04058392), the effects of receiving the polyphenol-rich Brazilian fruit Camu Camu in combination with antiretroviral therapy on Camu Camu tolerance, changes in inflammation and gut barrier markers, and microbe composition of HIV patients are being evaluated. Multi-factorial outcomes of polyphenol presence including promotion of mucus secretion, scavenging of free oxygen radicals in the intestinal lumen and exertion of antimicrobial effects on other mucin-degrading bacteria, have all been proposed in attempts to explain the “prebiotic-like” effects of polyphenols on *A*. *muciniphila* [40]. However, no published *in vitro* culture data on the biochemistry of polyphenol utilization in this organism is available currently, and hence the mechanism/s of polyphenolic stimulation of this organism have remained unclear. In this study we provide metabolic evidences demonstrating for the first time to our knowledge that *A*. *muciniphila* can co-metabolize EGCG together with either mucin or glucose (Figs 1–5; Tables 1, 2, S1 and S2) in the presence of mineral solutions and amino acids for EGCG and natural protein sources for glucose. The co-metabolism of EGCG with both mucin and glucose and its growth-stimulating effect on *A*. *muciniphila* here provides an important addition to the currently available proposed mechanisms of polyphenolic prebiotic effects on *A*. *muciniphila*. Certainly *A*. *muciniphila* would have ready access *in vivo* to mucin in the mucus layer and to glucose arising from fermentation of dietary fibers like ß-glucan and resistant starch [41] by other intestinal populations.

Efficient co-metabolism of EGCG with both mucin and glucose by *A*. *muciniphila* suggests that the protocooperative benefit of EGCG (polyphenol) and glucose-based dietary fibers may provide an effective way to enhance the relative abundance of this organism in the intestinal microbiota. Polyphenols and fiber are often consumed together, and the interaction between these two functional food groups, and the resultant positive impacts on the colonic bacteria and subsequent host health are increasingly being recognized [42], and should pave the way towards designing foods with optimal health benefits. It has been claimed that fermentation products from dietary fibers may enhance selectively the activities of certain bacterial populations, which, in turn may promote the growth of polyphenol-catabolizing bacteria [43]. Moreover, polyphenols may also influence the fermentation of such fibers by exhibiting both anti-microbial and prebiotic effects [28]. Thus, EGCG co-metabolism with glucose serves as a clear demonstration of how interactions between polyphenols and fibrous foods may enhance the probiotic attributes of *A*. *muciniphila*.

EGCG conversion by intestinal microbiota is well documented [44]. However, little information is available as to which of the individual intestinal bacterial populations are responsible. Our metabolomic data (Figs 2 and 4; Tables 1, 2 and S2) show that *A*. *muciniphila* can metabolize EGCG effectively to gallic acid and epigallocatechin (EGC) through ester hydrolysis. It can also isomerize EGCG to (-)-epicatechin gallate, which is then converted further to gallic acid and (-)-epicatechin (EC), again following ester hydrolysis. This finding is in line with data from animal gut studies, where EGCG has been reported to be hydrolyzed to EGC and gallic acid by the intestinal microbiota [39]. The metabolic pathway of EGCG degradation by *A*. *muciniphila* described here differs from those reported for the intestinal microbiota [44]. For example, we found that *A*. *muciniphila* can isomerize EGC to (+)-gallocatechin. The latter may then be further metabolized, leading to production of hydroxyhydroquinone. The situation reported for the intestinal microbiota suggests that the EGC and EC may be degraded into 5-(3′,4′,5′-trihydroxyphenyl)-γ-valerolactone [45], which are further metabolized into low molecular weight C6−C1 phenolic and aromatic acids that eventually enter the blood circulation system and are finally excreted in urine [46].

We are aware that *in vivo* environmental conditions are quite different to those used here, and so such differences in the metabolic fates of EGCG are to be expected. Animal model studies are now needed to confirm the stimulatory effects of EGCG co-metabolism with mucin and especially with glucose on *A*. *muciniphila* as well as its subsequent preventive influences on metabolic diseases. We are also aware that although many experimental and clinical studies have shown a positive association between abundance of *A*. *muciniphila* and metabolic diseases including HIV [5] and several cancers [8–10,39], and high abundances of *A*. *muciniphila* have also been associated with colitis and metabolic syndrome [47] and pancreatitis in mice [48]. As *A*. *muciniphila* consists of four phylogroups [49,50], each may differ in its functional properties of GI tract colonization and host function modulation, including oxygen tolerance, adherence to epithelial cells, iron and sulfur metabolism, and bacterial aggregation [51]. Further studies are required to see whether this is the case.

## Supporting information

S1 Table EGCG concentrations in the mucin BS broths and glucose supplemented modified BS broths of *Akkermansia muciniphila*. (PDF)

S2 Table Polyphenolic compounds and their relative concentrations identified in cell extracts of *Akkermansia muciniphila* with HPLC using standard compounds. (PDF)

## References

[1] Collado MC, Derrien M, Isolauri E, de Vos WM, Salminen S. Intestinal integrity and *Akkermansia muciniphila*, a mucin-degrading member of the intestinal microbiota present in infants, adults, and the elderly. Appl Environ Microbiol. 2007; 73:7767–70. doi: 10.1128/AEM.01477-07 . 17933936 PMC2168041

[2] Everard A, Belzer C, Geurts L, Ouwerkerk JP, Druart C, Bindels LB, et al. Cross-talk between *Akkermansia muciniphila* and intestinal epithelium controls diet-induced obesity. Proc Natl Acad Sci USA. 2013; 110:9066–9071. doi: 10.1073/pnas.1219451110 23671105 PMC3670398

[3] van Passel MWJ, Kant R, Zoetendal EG, Plugge CM, Derrien M, Malfatti SA, et al. The genome of *Akkermansia muciniphila*, a dedicated intestinal mucin degrader, and its use in exploring intestinal metagenomes. PLoS One. 2011; 6:e16876. doi: 10.1371/journal.pone.0016876 . 21390229 PMC3048395

[4] Kosciow K, Deppenmeier U. Characterization of three novel beta-galactosidases from *Akkermansia muciniphila* involved in mucin degradation. Int J Biol Macromol. 2020; 149:331–340. doi: 10.1016/j.ijbiomac.2020.01.246 . 31991210

[5] Ouyang J, Lin L, Isnard S, Fombuena B, Peng XR, Marette A, et al. Bacterium *Akkermansia muciniphila*: a sentinel for gut permeability and its relevance to HIV-related inflammation. Front Immunol. 2020; 11:645. doi: 10.3389/fimmu.2020.00645 . 32328074 PMC7160922

[6] Plovier H, Everard A, Druart C, Depommier C, van Hul M, Geurts C, et al. A purified membrane protein from *Akkermansia muciniphila* or the pasteurized bacterium improves metabolism in obese and diabetic mice. Nat Medicine. 2017; 23:107–113. doi: 10.1038/nm.4236 . 27892954

[7] Earley H, Lennon G, Balfe A, Coffey JC, Winter DC, O’Connell PR. The abundance of *Akkermansia muciniphila* and its relationship with sulphated colonic mucins in health and ulcerative colitis. Front Immunol. 2019; 9:15683. doi: 10.1038/s41598-019-51878-3 . 31666581 PMC6821857

[8] Weir TL, Manter DK, Sheflin AM, Barnett BA, Heuberger AL, Ryan EP. Stool microbiome and metabolome differences between colorectal cancer patients and healthy adults. PLoS One. 2013; 8:e70803. doi: 10.1371/journal.pone.0070803 . 23940645 PMC3735522

[9] Routy B, Chatelier EL, Derosa L, Duong CPM, Alou MT, Daillère R, et al. Gut microbiome influences efficacy of PD-1 based immunotherapy against epithelial tumors. Science. 2018; 359:91–97. doi: 10.1126/science.aan3706 . 29097494

[10] Derosa L, Routy B, Fidelle M, Lebba V, Alla L, Pasolli E, et al. Gut bacteria composition drives primary resistanceto cancer immunotherapy in renal cell carcinoma patients. Eur Urol. 2020; 78:195–206. doi: 10.1016/j.eururo.2020.04.044 . 32376136

[11] Cani PD, de Vos WM. Next-generation beneficial microbes: the case of *Akkermansia muciniphila*. Front Microbiol. 2017; 8:1765. doi: 10.3389/fmicb.2017.01765 . 29018410 PMC5614963

[12] Derrien M, Belzer C, de Vos WM. *Akkermansia muciniphila* and its role in regulating host functions. Microb Pathogenesis. 2017; 106:171–181. doi: 10.1016/j.micpath.2016.02.005 . 26875998

[13] Macchione IG, Lopetuso LR, Ianiro G, Napoli M, Gibiino G, Rizzatti G, et al. *Akkermansia muciniphila*: key player in metabolic and gastrointestinal disorders. Eur Rev Med Pharmacol Sci. 2109; 23:8075–8083. doi: 10.26355/eurrev_201909_19024 . 31599433

[14] Anhê FF, Pilon G, Roy D, Desjardins Y, Levy E, Marette A. Triggering *Akkermansia muciniphila* with dietary polyphenols: A new weapon to combat the metabolic syndrome? Gut Microbes. 2016; 7:146–153. doi: 10.1080/19490976.2016.1142036 . 26900906 PMC4856456

[15] Kemperman RA, Gross G, Mondot S, Possemiers S, Marzorati M, van de Wiele T, et al. Impact of polyphenols from black tea and red wine/grape juice on a gut model microbiome. Food Res Int. 2013; 53:659–669. 10.1016/j.foodres.2013.01.034.

[16] Anhê FF, Roy D, Pilon G, Dudonnê S, Matamoros S, Varin TV, et al. A polyphenol-rich cranberry extract protects from diet-induced obesity, insulin resistance and intestinal inflammation in association with increased *Akkermansia* spp. population in the gut microbiota of mice. Gut. 2015; 64:872–883. doi: 10.1136/gutjnl-2014-307142 . 25080446

[17] Roopchand DE, Carmody RN, Kuhn P, Moskal K, Rojas-Silva P, Turnbaugh PJ, et al. Dietary polyphenols promote growth of the gut bacterium *Akkermansia muciniphila* and attenuate high-fat diet–induced metabolic syndrome. Diabetes. 2015; 64:2847–2858. doi: 10.2337/db14-1916 . 25845659 PMC4512228

[18] Zhang NN, Guo WH, Han H, Zhou R, Liu QP, Zheng BD, et al. Effect of a polyphenol-rich *Canarium album* extract on the composition of the gut microbiota of mice fed a high-fat diet. Molecules. 2018; 23:2188. doi: 10.3390/molecules23092188 . 30200213 PMC6225199

[19] Xia Y, Tan DH, Akbary R, Kong J, Seviour R, Kong YH. Aqueous raw and ripe Pu-erh tea extracts alleviate obesity and alter cecal microbiota composition and function in diet-induced obese rats. Appl Microbiol Biotechnol. 2019; 103:1823–1835. doi: 10.1007/s00253-018-09581-2 . 30610284

[20] Manach C, Scalbert A, Morand C, Rémésy C, Jiménez L. Polyphenols: Food sources and bioavailability. Am J Clin Nutr. 2004; 79:727–747. doi: 10.1093/ajcn/79.5.727 . 15113710

[21] Loo YT, Howell K, Chan MI, Zhang PZ, Ng K. Modulation of the human gut microbiota by phenolics and phenolic fiber-rich foods. Compr Rev Food Sci Food Saf. 2020; 19:1268–1298. doi: 10.1111/1541-4337.12563 . 33337077

[22] Yang QQ, Wei XL, Fang YP, Gan RY, Wan M, Ge YY, et al. Nanochemoprevention with therapeutic benefits: An updated review focused on epigallocatechin gallate delivery. Crit Rev Food Sci Nutr. 2020; 60:1243–1264. doi: 10.1080/10408398.2019.1565490 . 30799648

[23] Chang Y, Yang Y, Xu N, Mu H, Zhang H. Improved viability of *Akkermansia muciniphila* by encapsulation in spray dried succinate-grafted alginate doped with epigallocatechin-3-gallate. Int J Biol Macromol. 2020; 159:373–382. doi: 10.1016/j.ijbiomac.2020.05.055 . 32422255

[24] Sheng L, Jena PK, Liu HX, Hu Y, Nagar N, Bronner DN, et al. Obesity treatment by epigallocatechin-3-gallate–regulated bile acid signaling and its enriched *Akkermansia muciniphila*. FASEB J. 2018; 32:fj201800370R. doi: 10.1096/fj.201800370R . 29882708 PMC6219838

[25] Ushiroda C, Naito Y, Takagi T, Uchiyama K, Mizushima K, Higashimura Y, et al. Green tea polyphenol (epigallocatechin-3-gallate) improves gut dysbiosis and serum bile acids dysregulation in high-fat diet-fed mice. J Clin Biochem Nutr. 2019; 65:34–46. doi: 10.3164/jcbn.18-116 . 31379412 PMC6667385

[26] Dey P, Olmstead BD, Sasaki GY, Vodovotz Y. Epigallocatechin gallate but not catechin prevents nonalcoholic steatohepatitis in mice similar to green tea extract while differentially affecting the gut microbiota. J Nutr Biochem. 2020; 84:108455. doi: 10.1016/j.jnutbio.2020.108455 . 32688217

[27] Liu X, Zhao K, Jing N, Zhao Y, Yang X. EGCG regulates fatty acid metabolism of high-fat diet-fed mice in association with enrichment of gut *Akkermansia muciniphila*. J Funct Foods. 2020; 75:104265. 10.1016/j.jff.2020.104261.

[28] Edwards CA, Havlik J, Cong W, Mullen W, Preston T, Morrison DJ, et al. Polyphenols and health: interactions between fibre, plant polyphenols and the gut microbiota. Nutr Bull. 2017; 42:356–360. doi: 10.1111/nbu.12296 . 29200959 PMC5698720

[29] Derrien M, Vaughan EE, Plugge CM, de Vos WM. *Akkermansia muciniphila* gen. nov., sp. nov., a human intestinal mucin-degrading bacterium. Int J Syst Evol Microbiol. 2004; 54:1469–1476. doi: 10.1099/ijs.0.02873-0 . 15388697

[30] Miller RS, Hoskins LC. Mucin degradation in human colon ecosystems. Fecal population densities of mucin-degrading bacteria estimated by a ‘most probable number’ method. Gastroenterology. 1981; 81:759–765. 10.1172/JCI111795. 7262520

[31] Stams AJM, van Dijk JB, Dijkema C, Plugge CM. Growth of syntrophic propionate-oxidizing bacteria with fumarate in the absence of methanogenic bacteria. Appl Environ Microbiol. 1993. 59:1114–1119. doi: 10.1128/aem.59.4.1114-1119.1993 . 16348912 PMC202247

[32] Delaney ML, Onderdonk AB. Evaluation of the anaeropack system for growth of clinically significant anaerobes. J Clinical Microbiol. 1997; 35:558–562. doi: 10.1128/jcm.35.3.558-562.1997 . 9041388 PMC229626

[33] Crittenden R, Karppinen S, Ojanen S, Tenkanen M, Fagerström R, Mättö J. *In vitro* fermentation of cereal dietary fibre carbohydrates by probiotic and intestinal bacteria. J Sci Food Agric. 2002; 82:781–789. doi: 10.1080/15287394.2011.615106 . 22047159

[34] Kong YH, He ML, McAlister T, Seviour R, Forster R. Quantitative fluorescence in situ hybridization of microbial communities in the rumens of cattle fed different diets. Appl Environ Microbiol. 2010; 76:6933–6938. doi: 10.1128/AEM.00217-10 . 20802069 PMC2953036

[35] Haug K, Cochrane K, Nainala VC, Williams M, Chang J, Jayseelan KV, et al. MetaboLights: a resource evolving in response to the needs of its scientific community. Nuc Acid Res. 2020; 48:D440–D444. doi: 10.1093/nar/gkz1019 . 31691833 PMC7145518

[36] Derrien M, Collado MC, Ben-Amor K, Salminen S, de Vos MM. The mucin degrader *Akkermansia muciniphila* is an abundant resident of the human intestinal tract. Appl Environ Microbiol. 2008; 74:1646–1648. doi: 10.1128/AEM.01226-07 . 18083887 PMC2258631

[37] Rothwell JA, Pérez-Jiménez J, Neveu V, Medina-Ramon A, M’Hiri N, Garcia-Lobato P, et al. Phenol-Explorer 3.0: a major update of the Phenol-Explorer database to incorporate data on the effects of food processing on polyphenol content. Database bat070. 2013; doi: 10.1093/database/bat070 . 24103452 PMC3792339

[38] Isnard S, Fombuena B, Ouyan J, Varin TV, Richard C, Marette A, et al. Repurposing metformin in nondiabetic people with HIV: influence on weight and gut microbiota. Open Forum Infect Dis. 2020; 7:ofaa338. doi: 10.1093/ofid/ofaa338 . 32964062 PMC7489545

[39] Grajeda-Iglesias C, Durand S, Daillére R, Iribarren K, Lemaitre F, Derosa L, et al. Oral administration of *Akkermansia muciniphila* elevates systemic antiaging and anticancer metabolites. Aging. 2021; 13:6375–6390. doi: 10.18632/aging.202739 . 33653967 PMC7993698

[40] Anhê FF, Varin TV, Le Barz M, Desjardins Y, Levy E, Roy D, et al. Gut microbiota dysbiosis in obesity-linked metabolic diseases and prebiotic potential of polyphenol–rich extracts. Curr Obes Rep. 2015; 4:389–400. doi: 10.1007/s13679-015-0172-9 . 26343880

[41] Lattimer JM, Haub MD. Effects of dietary fiber and its components on metabolic health. Nutrients. 2010; 2:1266–1289. doi: 10.3390/nu2121266 . 22254008 PMC3257631

[42] van Hul M, Cani PD. Targeting carbohydrates and polyphenols for a healthy microbiome and healthy weight. Curr Nutr Rep. 2019; 8:307–316. doi: 10.1007/s13668-019-00281-5 . 31161579 PMC6904403

[43] Tzounis X, Vulevic J, Kuhnle GGC, Geoege T, Spencer JPE. Flavanol monomer-induced changes to the human faecal microflora. Br J Nutr. 2008; 99:782–792. doi: 10.1017/S0007114507853384 . 17977475

[44] Xing LJ, Zhang H, Qi RL, Tsao R, Mine Y. Recent advances in the understanding of the health benefits and molecular mechanisms associated with green tea polyphenols. J Agric Food Chem. 2019; 67:1029–1043. doi: 10.1021/acs.jafc.8b06146 . 30653316

[45] Li C, Lee MJ, Sheng S, Meng X, Prabhu S, Winnik B, et al. Structural identification of two metabolites of catechins and their kinetics in human urine and blood after tea ingestion. Chem Res Toxicol. 2000; 13:177–184. doi: 10.1021/tx9901837 . 10725114

[46] Unno K, Pervin M, Nakagawa A, Iguchi K, Hara A, Takagaki A, et al. Blood−brain barrier permeability of green tea catechin metabolites and their neuritogenic activity in human neuroblastoma Sh-Sy5y cells. Mol Nutr Food Res. 2017; 61:1700294. doi: 10.1002/mnfr.201700294 . 28891114

[47] Chassaing B, Koren O, Goodrich JK, Poole AC, Srinivasan S, Ley RE, et al. Dietary emulsifiers impact the mouse gut microbiota promoting colitis and metabolic syndrome. Nature. 2015; 519:92–96. doi: 10.1038/nature14232 . 25731162 PMC4910713

[48] van den Berg FF, Hugenholtz F, Boermeester MA, Zaborina O, Alverdy JC. Spatioregional assessment of the gut microbiota in experimental necrotizing pancreatitis. BJS Open. 2021; 5:zarab061. doi: 10.1093/bjsopen/zrab061 . 34518874 PMC8438261

[49] Guo X, Li S, Zhang J, Wu F, Li X, Wu D, et al. Genome sequencing of 39 *Akkermansia muciniphila* isolates reveals its population structure, genomic and functional diverisity, and global distribution in mammalian gut microbiotas. BMC Genomics. 2017; 18:1–12. doi: 10.1186/s12864-016-3406-7 . 29047329 PMC5648452

[50] Kirmiz N, Galindo K, Cross KL, Luna E, Rhoades N, Podar M, et al. Comparative genomics guides elucidation of vitamin B 12 biosynthesis in novel human-associated *Akkermansia* strains. Appl Environ Microbiol. 2019; 86:e02117–19. doi: 10.1128/AEM.02117-19 . 31757822 PMC6974653

[51] Becken B, Davey L, Middleton DR, Mueller KD, Sharma A, Holmes Z, et al. Genotypic and phenotypic diversity among human isolates of *Akkermansia muciniphila*. mBio. 2021; 12:eoo478–21. doi: 10.1128/mBio.00478-21 . 34006653 PMC8262928

