# Supplementary material for: In vitro co-metabolism of epigallocatechin-3-gallate (EGCG) by the mucin-degrading bacterium Akkermansia muciniphila
Source: PLoS One. 2021 Dec 2;16(12):e0260757. doi: 10.1371/journal.pone.0260757 (PMC8638859; doi:10.1371/journal.pone.0260757)
Supplement: S1 Table — (PDF) [file pone.0260757.s001.pdf]

S1 Table. EGCG concentrations in the mucin BS broths and glucose supplemented modified BS broths of *Akkermansia muciniphila*.

Experimental setup	EGCG concentration (mg L^{-1})		
	Start[a]	End	Incubation time (h)
BS[b]+mucin+EGCG (150 mg L^{-1})	150	13.2 (± 2.7)[c]	24
BS–mucin+EGCG (150 mg L^{-1})	150	146.5 (± 5.1)	24, 48, 72
BS+mucin+EGCG (350 mg L^{-1})	350	56.5 (± 3.2)	24
BS–mucin+EGCG (350 mg L^{-1})	350	286.3 (± 8.4)	24, 48, 72
BS+mucin+EGCG (500 mg L^{-1})	500	138.9 (± 6.3)	24
BS–mucin+EGCG (500 mg L^{-1})	500	491.2 (± 8.5)	24, 48, 72
Modified BS+Glu[d]+EGCG (150 mg L^{-1})	150	17.3 (± 3.5)	24
Modified BS–Glu+EGCG (150 mg L^{-1})	150	146.7 (± 4.9)	24, 48, 72
Modified BS+Glu+EGCG (350 mg L^{-1})	350	76.1 (± 5.3)	24
Modified BS–Glu+EGCG (350 mg L^{-1})	350	345.8 (± 5.5)	24, 48, 72
Modified BS+Glu+EGCG (500 mg L^{-1})	500	157.5 (± 5.1)	24
Modified BS+Glu+EGCG (500 mg L^{-1})	500	490.3 (± 7.4)	24, 48, 72

[a] Theoretical EGCG concentrations supplemented at the beginning of the experiments

[b] BS represents the BS broth of Derrien et al. (2004)

[c] Values represent means of 6 replicate experiments and their standard deviations

[d] Glu represent glucose supplemented at 2 g L^{-1} final concentration.
