# Supplementary material for: In vitro co-metabolism of epigallocatechin-3-gallate (EGCG) by the mucin-degrading bacterium Akkermansia muciniphila
Source: PLoS One. 2021 Dec 2;16(12):e0260757. doi: 10.1371/journal.pone.0260757 (PMC8638859; doi:10.1371/journal.pone.0260757)
Supplement: S2 Table — (PDF) [file pone.0260757.s002.pdf]

S2 Table. Polyphenolic compounds and their relative concentrations identified in cell extracts of *Akkermansia muciniphila* with HPLC using standard compounds.

**Polyphenolic standards**	**Relative concentrations (mg L^{-1}) of EGCG metabolites identified in the cell extracts of *A. muciniphila* incubated under different conditions**	
	Mucin BS broth with EGCG (350 mg L^{-1})	Glucose supplemented modified BS broth supplimented with EGCG (350 mg L^{-1})
**Hydroxyhydroquinone**	1.9 (± 0.2)[a]	10.4 (± 0.7)
**Gallic acid**	89.5 (± 4.2)	57.1 (± 2.3)
**Gallocatechin**	0	32.4 (± 1.3)
**(-)-Epigallocatechin**	0	0
**3,4-Dihydroxybenzaldehyde**	0	0
**(-)-Catechin gallate**	97.6 (± 4.2)	26.8 (± 2.2)

[a] mean value and their standard deviation of a polyphenolic compound from 6 replicate samples of the cell extracts of *A. muciniphila* incubated under a certain condition
